# Association between estimated glucose disposal rate and metabolic-associated fatty liver disease among US adults: A cross-sectional study

**DOI:** 10.1097/MD.0000000000045652

**Published:** 2025-10-31

**Authors:** Rong He, Kecen Zhao

**Affiliations:** a Department of Clinical Laboratory Medicine, The First Affiliated Hospital (Southwest Hospital) of Army Medical University, Chongqing, China.

**Keywords:** body adipose distribution, estimated glucose disposal rate, insulin resistance, metabolic-associated fatty liver disease, NHANES

## Abstract

The association between estimated glucose disposal rate (eGDR) and metabolic-associated fatty liver disease (MAFLD) is currently lacking. This study aimed to investigate the relationship between eGDR-related indices and the prevalence of MAFLD. The study analyzed data from the 2017 to 2018 cycle of the National Health and Nutrition Examination Survey. Multivariate logistic regression models were employed to examine the relationship between eGDR-related indices and MAFLD. Receiver operating characteristic curves were used to compare the diagnostic validity of eGDR-related indices and other insulin resistance indicies. A total of 2273 participants were included. In the fully adjusted multivariate logistic regression model, eGDR-waist-hip ratio (OR, 0.65; 95% CI, 0.58–0.72; *P* <.001), eGDR-waist circumference (WC) (OR, 0.62; 95% CI, 0.56–0.67; *P* <.001), eGDR-body mass index (BMI) (OR, 0.62; 95% CI, 0.56–0.67; *P* <.001), and lnGDR (OR, 0.76; 95% CI, 0.66–0.88; *P* <.01) were negatively associated with MAFLD. Mediation analysis indicated that android: gynoid ratio played a role in this association, explaining 28.8%, 17.2%, 18.6%, and 33.6% of the associations, respectively (all *P* for mediation <.001). Receiver operating characteristic curves found that eGDR-WC (areas under curves, 0.794; 95% CI, 0.776–0.813) and eGDR-BMI (areas under curves, 0.791; 95% CI, 0.772–0.810) had greater diagnostic validity for MAFLD. The present study suggests a potential inverse association between eGDR and MAFLD. In addition, eGDR-WC and eGDR-BMI could potentially serve as useful screening metrics for identifying populations at early risk of MAFLD.

## 1. Introduction

Nonalcoholic fatty liver disease (NAFLD) is prevalent globally, affecting 25 to 35% of the population, and its incidence is rising across all age groups worldwide.^[1–4]^ Recognizing the limitations of the term NAFLD, which relies on exclusion criteria and lacks reflection of the underlying pathophysiology, the new term metabolic-associated fatty liver disease (MAFLD) was introduced in 2020.^[5]^ In contrast to NAFLD, MAFLD identifies more patients with metabolic abnormalities and hepatic steatosis, enhancing its clinical applicability.^[6]^ Given the rising global prevalence of obesity and diabetes, the MAFLD pandemic may worsen in the future.^[7]^ Therefore, accurately identifying underlying MAFLD patients as early as possible is essential for improving their prognosis.

Factors associated with MAFLD pathogenesis include obesity, abnormal body adipose distribution, insulin resistance (IR), and so on.^[8–10]^ The trunk has 2 types of fat depots, the gynoid adipose distribution (also called pear-shaped), characterized by increased fat in the hip and thigh areas, and the android pattern characterized by increased fat in the trunk (also called apple-shaped).^[11]^ While both distributions could pose significant health risks, but to varying degrees. Certain studies have revealed that a gynoid adipose distribution is associated with a decreased risk of metabolic and cardiovascular diseases than an android pattern.^[12]^ Ciardullo et al have found a higher android: gynoid ratio (A: G ratio) was associated with an increased prevalence of NAFLD.^[13]^ IR plays a crucial role in the pathogenesis of MAFLD, involving reduced insulin sensitivity in peripheral tissues, which manifests as impaired glucose uptake and oxidation.^[14–16]^ Interventions targeting IR have been reported to effectively improve hepatic steatosis in persons with MAFLD.^[17,18]^

The gold standard for assessing IR, the euglycemic-hyperinsulinemic clamp study, has limited clinical utility due to its invasive and time-consuming nature. Homeostasis model assessment of IR (HOMA-IR) is a commonly used method for assessing IR, however, its utility is limited in individuals undergoing insulin treatment or with beta cell dysfunction.^[19]^ Previous studies have introduced another surrogate indicator, the triglyceride-glucose (TyG) index, while the sensitivity and specificity of the TyG index appeared inadequate in certain conditions.^[20]^ Recently, estimated glucose disposal rate (eGDR) has been introduced as a marker that correlated well with IR, incorporating parameters such as waist circumference (WC), blood pressure status, and glycated hemoglobin (HbA1c) level.^[21]^ It has been validated for assessing insulin sensitivity in patients with diabetes mellitus.^[22]^ Additionally, researchers have developed various eGDR models utilizing metrics such as WC, HbA1c, body mass index (BMI), waist-to-hip ratio, and diastolic blood pressure (DBP), these methods allowed an estimation of IR compared with the gold standard test.^[23,24]^

Previous studies have demonstrated that eGDR could predict the onset of metabolic diseases in clinical settings. A cross-sectional study involving 112 patients with type 1 diabetes mellitus found that eGDR exhibited a high sensitivity and specificity for diagnosing metabolic syndrome in this patient group.^[25]^ Similarly, a prospective study from the Netherlands revealed a statistically significant association between eGDR and NAFLD presence.^[26]^ However, studies exploring the relationship between eGDR and MAFLD are currently lacking. Therefore, we carried out an observational study analyzing the National Health and Nutrition Examination Survey (NHANES) database to delve into the potential association between eGDR-related indices and MAFLD.

## 2. Materials and methods

### 2.1. Study population

This study used data from the NHANES, a cross-sectional survey designed to assess the health and nutritional status of both adults and children in the US. Since 1999, it has been revised biennially and received approval from the National Center for Health Statistics Research Ethics Review Board. Informed consent was obtained from all participants.^[27]^ In this study, we used 1 NHANES 2-year cycle (2017–2018), since it provides database for liver fibrosis assessment by FibroScan®.

Participants were selected from 4 cycles of NHANES, from 2017 to 2018, aged above 20 years, and weighted to reflect the noninstitutionalized civilian population of the United States. Following the exclusion of participants with missing data on eGDR-related indices, liver steatosis assessment, and other covariates, the eligible participants were included in the study. Figure S1, Supplemental Digital Content, https://links.lww.com/MD/Q547 illustrates the participant inclusion and exclusion procedures applied in this study. The standardized difference analysis in Table S1, Supplemental Digital Content, https://links.lww.com/MD/Q547 confirms that the included and excluded groups were comparable across most baseline characteristics (standardized difference ≤ 10%), indicating minimal bias from missing data.

### 2.2. Calculation of IR-related indices

eGDR (mg × kg^−1^ × min^−1^) was used to measure IR. HbA1c was measured at baseline when the participants provided their blood samples. Body weight, height, blood pressure, waist and hip circumference were obtained when people participated in the physical examinations at a mobile examination center. Hypertension was defined as systolic blood pressure (≥130 mm Hg), DBP (≥85 mm Hg), or a hypertension diagnosis or treatment with antihypertensive drugs. eGDR-WHR, eGDR-WC, eGDR-BMI, and the glucose disposal rate of the natural logarithm (lnGDR) were calculated according to the following formulas^[23,24]^:


$$\begin{array}{l} \begin{array}{lll} & eGDR-WHR=24.31-(12.22\times WHR)\; & -(3.29\times HT)-(0.57\times HbA1c)\; \end{array} \end{array}$$

$$\begin{array}{l} \begin{array}{lll} & eGDR-WC=21.16-(0.09\times WC)\; & -(3.41\times HT)-(0.55\times HbA1c)\; \end{array} \end{array}$$

$$\begin{array}{l} \begin{array}{lll} & eGDR-BMI=19.02-(0.22\times BMI)\; & -(3.26\times HT)-(0.61\times HbA1c)\; \end{array} \end{array}$$

$$\begin{array}{l} \begin{array}{lll} & InGDR=4.964-(0.121\times HbA1c)\; & -(0.012\times DBP)-(1.409\times WHR)\; \end{array} \end{array}$$

$$\begin{array}{l} BMI=Weight\;\left(kg\right)\;/\;Height\;{\left(m\right)}^{2} \end{array}$$

In these formulas, WHR= waist-hip ratio (%); HT = hypertension (yes = 1/no = 0); HbA1c = HbA1c (%); WC = waist circumference (cm); BMI = body mass index (kg/m^2^); DBP = DBP (mm Hg).

TyG index and HOMA − IR were calculated according to the following formulas^[28,29]^:


$$\begin{array}{l} \begin{array}{lll} & TyG=In\;[Fasting\;triglycerides\;(mg/dL)\;\; & \times \;Fasting\;glucose\;\left(mg/dL\right)÷2\;]\;\; \end{array} \end{array}$$

$$\begin{array}{l} \begin{array}{lll} & HOMA-IR=Fasting\;glucose\;\left(mmol/L\right)\; & \times Fasting\;insulin\;(\mu U/mL)÷22.5\; \end{array} \end{array}$$

Triglyceride value is obtained from the standard battery of biochemical assessments. Ultraviolet rays In vitro test for the quantitative determination of fasting glucose. HbA1c in whole blood specimens is measured by non-porous ion exchange, high performance liquid chromatography and microcomputer technology. The fasting insulin is measured by 2-site immunoenzyme assay.

### 2.3. Assessment of MAFLD

Liver steatosis was defined as the controlled attenuation parameter score ≥285 dB/m.^[30]^ Based on BMI, participants were classified into 1 of 3 categories: normal BMI (18.5–24.9 kg/m^2^), overweight (25.0–29.9 kg/m^2^), or obese (≥30.0 kg/m^2^). MAFLD was defined based on hepatic steatosis and any of the 3 criteria listed below: overweight/obese, type 2 diabetes (fasting glucose ≥6.9 mmol/L, or previously diagnosed with diabetes, or taking insulin now), or metabolic dysregulation. Metabolic dysregulation is defined as the presence of at least 2 of the following: WC ≥102 cm for male and ≥88 cm for female; blood pressure ≥130/85 mm Hg or on antihypertensive therapy; plasma triglycerides ≥1.70 mmol/L or on lipid-lowering therapy; serum high-density lipoprotein cholesterol <1.0 mmol/L for male and <1.3 mmol/L for female or specific drug treatment; diabetes or prediabetes (fasting glucose level 5.6 to 6.9 mmol/L, or 2-h post-load glucose level 7.8 to 11.0 mmol or HbA1c 5.7% to 6.4%); serum hypersensitivity C-reactive protein levels >2 mg/L; HOMA-IR score ≥2.5.^[31]^

### 2.4. Parameters of body adipose distribution

Android and gynoid regions were defined using the Hologic APEX software. The android area was defined as the lower trunk area, bounded by the pelvic horizontal cut line on its lower edge and an upper line automatically placed by the software. The upper gynoid line was placed 1.5 times of the height of android region below the pelvic line and the lower gynoid line was placed such that the distance between the 2 gynoid lines was twice the height of the android region. All these lines were automatically placed by Hologic software.^[32]^

### 2.5. Covariates

The demographic variables included age, sex, race, poverty income ratio, and educational level. The questionnaire data included smoking status, alcohol status, and recreational physical activity level. Smoking status was categorized as never smoker and smoker. Alcoholic status was categorized as excessive alcohol (>1 drink per day for females, >2 drinks per day for males, 1 drink was defined as a 12 oz. beer, a 5 oz. glass of wine, or one and a half ounces of liquor) and non-excessive alcohol. Hypertension was characterized by a blood pressure of at least 130/85 mm Hg or the use of antihypertensive medication. Diabetes status was assessed based on glycated hemoglobin levels (≥6.5%), fasting plasma glucose levels (≥126 mg/dL), or a confirmed diagnosis or treatment with antidiabetic medication. The collection and measurement of triglycerides and cholesterol were conducted at the NHANES Mobile Examination Center.

### 2.6. Statistical analysis

To address potential biases, data with missing covariates were excluded, and baseline demographic characteristics of both included and excluded groups were compared using standardized differences, considering differences below 10% as negligible.^[33]^ (Table S1, Supplemental Digital Content, https://links.lww.com/MD/Q547). The baseline characteristics of the participants included in the study were summarized using median values and interquartile range (IQR) (continuous variables; presented as the median [IQR]) or proportions (categorical variables; expressed as N [%]). Due to the complex probability cluster design used in NHANES, all statistical analyses in this study accounted for weights. Weighted multivariable-adjusted logistic regression was used to evaluate the eGDR-related indices and MAFLD, with findings expressed as odds ratios (OR) and 95% confidence intervals (CI). Covariates for multivariable analysis were selected based on theoretical insights from existing literature, aligning with recommendations, rather than relying on statistical criteria.^[34]^ Model 1 did not include confounder adjustments. Model 2 incorporated adjustments for demographic covariates such as age, gender, race, marital status, and family income; Model 3 additionally adjusted for family income, activity level, educational level, alcoholic status, and smoking status, as these covariates have been regarded as the risk factors for hepatic steatosis.^[35,36]^ The variance inflation factor and tolerance were used to evaluate the multicollinearity among the variables in the models (variance inflation factor <10 and tolerance >0.1 were considered non-multicollinearity).

Multivariate-adjusted restricted cubic splines (RCS) analysis and 2-piecewise linear regression were utilized to explore the dose-response relationship of eGDR-related indices on MAFLD. Mediation analysis was developed to examine whether the associations between eGDR-related indices and MAFLD were mediated by the A: G ratio, as a surrogate for adipose distribution. The area under the receiver operating characteristic (ROC) curve was used to evaluate the diagnostic value of IR-related indices for MAFLD. DeLong test was used to compare the areas under ROC curves.^[37]^ Stratified analysis was conducted to assess the potential moderating effects of age, sex, race, poverty income ratio, education level, smoking status, activity level, and alcoholic status. All analyses employed R software (version 4.2.0), all *P*-values were 2-tailed, and a *P*-value <.05 was considered statistically significant.

## 3. Results

### 3.1. General characteristics of NHANES

Among 2273 eligible adults with complete data on eGDR-related indices and MAFLD, the mean (IQR) age was 45 (32, 59) years, and 1105 (50%) were females; 324 (9%) of participants were Mexican American, 500 (10%) of participants were non-Hispanic Black, and 848 (66%) of participants were non-Hispanic White. Among the included participants, 823 (36%) were defined as MAFLD. Table 1 presents a comparison of the baseline characteristics between individuals with and without MAFLD. Participants with MAFLD were more likely to be elderly, male, and obesity, engaged in less activity, more likely to smoke, and had lower levels of eGDR-related indices.

**Table 1:** Characteristics of the included participants from NHANES 2017–2018.

Characteristic	Overall, N = 2273*	Non-MAFLD, N = 1450*	MAFLD, N = 823*	*P*-value†
Age (year)	45 (32–59)	43 (30–57)	50 (35–61)	**<.001**
Sex (%)				
Female	1105 (50%)	764 (55%)	341 (42%)	**.002**
Male	1168 (50%)	686 (45%)	482 (58%)	
PIR	3.43 (1.79–5.00)	3.47 (1.76–5.00)	3.43 (1.85–5.00)	.800
Education level (%)				
<High school	324 (7.5%)	200 (7.2%)	124 (8.0%)	.600
≥High school	1949 (93%)	1250 (93%)	699 (92%)	
Race/Ethnicity (%)				
Mexican American	324 (9%)	155 (6.5%)	169 (14%)	**<.001**
Non-Hispanic Black	500 (10%)	359 (11%)	141 (7.2%)	
Non-Hispanic White	848 (66%)	533 (67%)	315 (65%)	
Other/multiracial	601 (15%)	403 (16%)	198 (14%)	
BMI (kg/m^2^)				
<25.0	592 (27%)	560 (40%)	32 (2.6%)	**<.001**
Overweight	726 (30%)	493 (32%)	233 (26%)	
Obesity	955 (43%)	397 (28%)	558 (72%)	
Waist circumference (cm)	99 (88, 111)	93 (83, 103)	111 (102, 121)	**<.001**
Activity level (%)				
No activities	1065 (41%)	620 (36%)	445 (52%)	**<.001**
Regular activities	1208 (59%)	830 (64%)	378 (48%)	
Smoking status (%)				
Never smoker	1251 (57%)	835 (59%)	416 (54%)	**.031**
Smoker	1022 (43%)	615 (41%)	407 (46%)	
Alcohol status (%)				
Excessive alcohol	1104 (50%)	717 (50%)	387 (49%)	.800
Non-excessive alcohol	1169 (50%)	733 (50%)	436 (51%)	–
Hypertension (%yes)	1150 (44%)	604 (34%)	546 (61%)	**<.001**
Diabetes (%yes)	342 (11%)	125 (5%)	217 (22%)	**<.001**
Triglycerides (mmol/L)	1.28 (0.87–1.91)	1.05 (0.78–1.64)	1.75 (1.23–2.50)	**<.001**
HDL (mmol/L)	1.34 (1.11–1.63)	1.45 (1.22–1.71)	1.22 (1.03–1.40)	**<.001**
C-reactive protein (mg/L)	1.7 (0.8–3.9)	1.3 (0.7–3.0)	2.9 (1.3–5.5)	**<.001**
HOMA-IR	2.22 (1.37–4.03)	1.74 (1.10–2.70)	4.16 (2.45–6.53)	**<.001**
eGDR-WHR (mg × kg^−1^ × min^−1^)	8.96 (6.16–10.42)	9.75 (7.02–10.77)	6.41 (5.36–9.16)	**<.001**
eGDR-WC (mg × kg^−1^ × min^−1^)	7.86 (5.38–9.96)	9.19 (6.60–10.58)	5.31 (3.91–7.76)	**<.001**
eGDR-BMI (mg × kg^−1^ × min^−1^)	8.00 (5.73–9.99)	9.08 (6.83–10.51)	5.68 (4.36–7.84)	**<.001**
lnGDR (mg × kg^−1^ × min^−1^)	−5.12 (−5.98 to −4.27)	−4.94 (−5.7 to −4.15)	−5.52 (−6.36 to −4.74)	**<.001**

Bold values indicate statistically significant differences (*P* < .05).

BMI = body mass index, eGDR = estimated glucose disposal rate, HDL = high-density lipoprotein cholesterol, HOMA-IR = homeostasis model assessment-insulin resistance, lnGDR = the glucose disposal rate of the natural logarithm, MAFLD = metabolic-associated fatty liver disease, PIR = ratio of family income to poverty, WC = waist circumference, WHR = waist-hip ratio.

* Median (IQR) for continuous; n (%) for categorical.

† Independent sample *t*-test for continuous variables and the χ2 test for categorical variables;

### 3.2. eGDR-related indices and MAFLD prevalence

Weighted multivariable logistic regression models were employed to explore the relationship between eGDR-related indices and MAFLD. Table 2 shows that in Model 3, after adjusting for multiple variables, a negative association between eGDR-related indices and MAFLD prevalence was observed. The OR for MAFLD was 0.55 (95% CI, 0.47–0.64) in the fourth quartile of eGDR-WHR compared to the reference group. Similarly, the OR for MAFLD was 0.50 (95% CI, 0.45–0.56) in the fourth quartile of eGDR-WC, 0.53 (95% CI, 0.47–0.58) in the fourth quartile of eGDR-BMI, and 0.80 (95% CI, 0.67–0.96) in the fourth quartile of lnGDR, all compared to the reference group. Trend analysis showed a significant trend toward decreased prevalence of MAFLD with increasing eGDR-related indices quartile (all *P* for trend <.01). These results were robust across all models. No multicollinearity existed between the independent variable and other variables.

**Table 2:** Association of eGDR-WHR, eGDR-WC, eGDR-BMI and lnGDR with MAFLD.

	MAFLD						
	Continuous		Q1*	Q2	Q3	Q4	*P*_trend_ †
	OR (95% CI)‡	Effect sizes	OR (95% CI)	OR (95% CI)	OR (95% CI)	OR (95% CI)	
eGDR-WHR							
Model 1§	0.67 (0.63–0.72)	−0.39	Ref (1.00)∥	0.77 (0.71–0.83)	0.73 (0.67–0.79)	0.57 (0.53–0.61)	**<.001**
Model 2	0.64 (0.59–0.70)	−0.45	Ref (1.00)	0.76 (0.70–0.82)	0.70 (0.64–0.77)	0.54 (0.50–0.59)	**<.001**
Model 3	0.65 (0.58–0.72)	−0.44	Ref (1.00)	0.76 (0.66–0.88)	0.71 (0.61–0.82)	0.55 (0.47–0.64)	**<.001**
eGDR-WC							
Model 1	0.64 (0.60–0.68)	−0.45	Ref (1.00)	0.69 (0.64–0.75)	0.66 (0.61–0.72)	0.51 (0.48–0.53)	**<.001**
Model 2	0.61 (0.57–0.66)	−0.49	Ref (1.00)	0.69 (0.64–0.75)	0.65 (0.59–0.71)	0.50 (0.47–0.53)	**<.001**
Model 3	0.62 (0.56–0.67)	−0.49	Ref (1.00)	0.70 (0.61–0.80)	0.65 (0.55–0.77)	0.50 (0.45–0.56)	**<.001**
eGDR-BMI							
Model 1	0.62 (0.58–0.67)	−0.47	Ref (1.00)	0.70 (0.64–0.76)	0.67 (0.61–0.73)	0.51 (0.48–0.55)	**<.001**
Model 2	0.61 (0.57–0.66)	−0.49	Ref (1.00)	0.70 (0.64–0.77)	0.66 (0.61–0.73)	0.52 (0.49–0.55)	**<.001**
Model 3	0.62 (0.56–0.67)	−0.48	Ref (1.00)	0.71 (0.60–0.83)	0.67 (0.57–0.79)	0.53 (0.47–0.58)	**<.001**
lnGDR							
Model 1	0.73 (0.65–0.81)	−0.32	Ref (1.00)	0.88 (0.81–0.95)	0.81 (0.74–0.89)	0.76 (0.70–0.83)	**<.001**
Model 2	0.75 (0.67–0.84)	−0.28	Ref (1.00)	0.89 (0.81–0.97)	0.83 (0.76–0.82)	0.79 (0.72–0.87)	**<.001**
Model 3	0.76 (0.66–0.88)	−0.27	Ref (1.00)	0.90 (0.75–1.27)	0.84 (0.71–1.00)	0.80 (0.67–0.96)	**.006**

Bold values indicate statistically significant differences (*P* < .05).

BMI = body mass index, CI = confidence interval, eGDR = estimated glucose disposal rate, lnGDR = the glucose disposal rate of the natural logarithm, MAFLD = metabolic-associated fatty liver disease, OR = odds ratio, WC = waist circumference, WHR = waist-hip ratio.

* Q, quartile.

† Tests for trends based on the variables containing the median values for each quartile.

‡ Data were listed as the weighted odd ratio estimates and 95% confidence intervals.

§ Model 1 was non-adjusted model. Model 2 was adjusted for adjusted for age, gender, and race. Model 3 further adjusted for family income, activity level, educational level, alcoholic status, and smoking status.

∥ Ref, reference.

### 3.3. RCS curve plotting and threshold effect analysis

**Figure 1.**
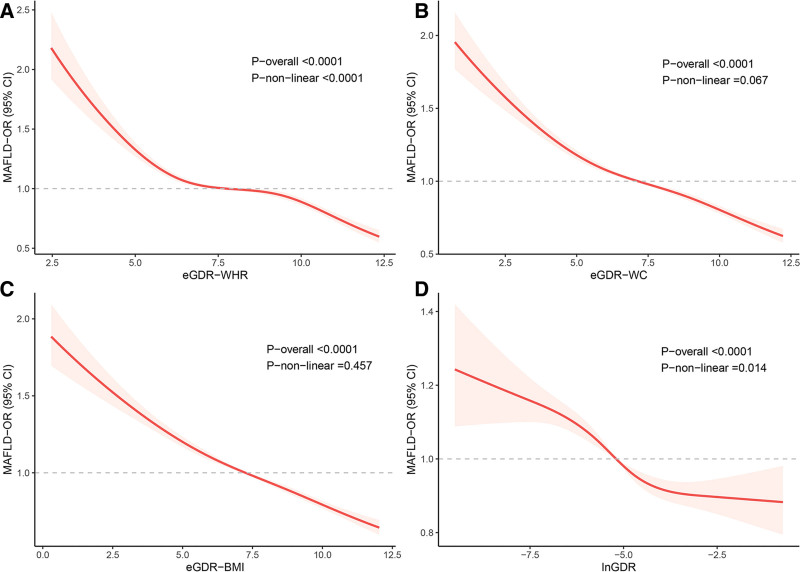
RCS plots of the association between eGDR-related indices and MAFLD prevalence. (A) eGDR-WHR; (B) eGDR-WC; (C) eGDR-BMI; (D) lnGDR. Adjusted for age, gender, race, family income, activity level, educational level, alcoholic status, and smoking status. BMI = body mass index, CI = confidence interval, eGDR = estimated glucose disposal rate, lnGDR = the glucose disposal rate of the natural logarithm, MAFLD = metabolic-associated fatty liver disease, OR = odds ratio, RCS = restricted cubic spline, WC = waist circumference, WHR = waist-hip ratio.

Figure 1 illustrates the relationship between eGDR-related indices and MAFLD in terms of dose-response. After multivariable adjustment, linear relationships were observed between eGDR-WC, eGDR-BMI and MAFLD (both *P* for nonlinear >.05). In contrast, significant nonlinear associations were found between eGDR-WHR and lnGDR with MAFLD (both *P* for nonlinear <.05) using RCS (both *P* for overall <.001). Based on the nonlinear relationship found in the RCS curve, a threshold effect analysis of eGDR-WHR and lnGDR on MAFLD was further performed by the 2-piecewise linear regression. As shown in Table 3, the inflection point of eGDR-WHR was 8.708 mg × kg^−1^ × min^−1^ (*P* for log-likelihood ratio <.001). Each unit increase of eGDR-WHR correlated with a 43% decrease in the prevalence of MAFLD below 8.708 mg × kg^−1^ × min^−1^, with a 75% decrease in the prevalence of MAFLD above this threshold. However, the threshold effect of lnGDR on MAFLD was not statistically significant (*P* for log-likelihood ratio >.05).

**Table 3:** Threshold effect analysis of eGDR-WHR and lnGDR on MAFLD by the 2-piecewise linear regression.

Inflection point	Adjusted OR (95% CI)	*P*-value
eGDR-WHR		
≤8.708	0.57 (0.44–0.74)	**.004**
>8.708	0.25 (0.16–0.42)	**.002**
Log-likelihood ratio	**<.001**	
LnGDR		
≤−3.735	0.70 (0.58–0.85)	**.007**
>−3.735	0.72 (0.45–1.15)	**.002**
Log-likelihood ratio	.092	

Adjusted for age, gender, race, family income, activity level, educational level, alcoholic status, and smoking status. Bold values indicate statistically significant differences (*P* < .05).

CI = confidence interval, eGDR = estimated glucose disposal rate, lnGDR = the glucose disposal rate of the natural logarithm, MAFLD = metabolic-associated fatty liver disease, OR = odds ratio, WHR = waist-hip ratio.

### 3.4. Mediation by adipose distribution

**Figure 2.**
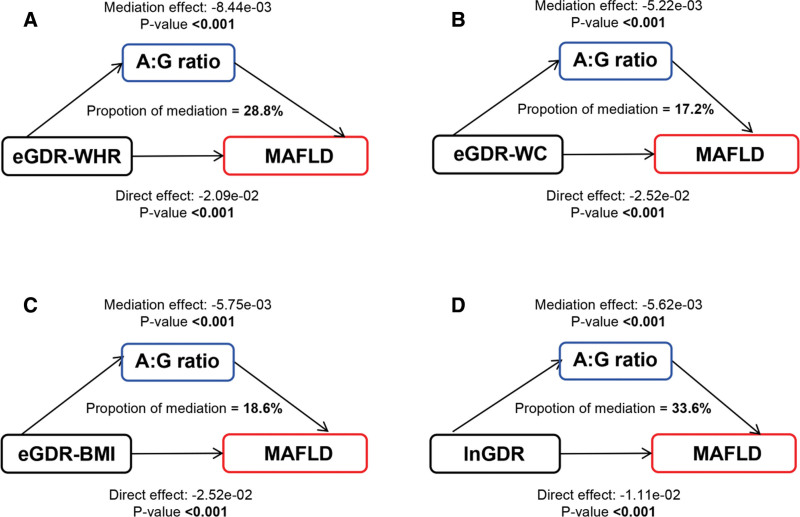
Path diagram of the mediation analysis of body adipose distribution between eGDR-related indices and MAFLD. A: G ratio = android: gynoid ratio, BMI = body mass index, eGDR = estimated glucose disposal rate, lnGDR = the glucose disposal rate of the natural logarithm, MAFLD = metabolic-associated fatty liver disease, WC = waist circumference, WHR = waist-hip ratio.

A significant negative relationship between eGDR-related indices and the A: G ratio was found by the weighted multiple linear regression model (Table S2, Supplemental Digital Content, https://links.lww.com/MD/Q547). As shown in Figure 2, mediation analyses revealed that the A: G ratio partially mediated the associations between eGDR-WHR, eGDR-WC, eGDR-BMI, lnGDR, and MAFLD, explaining 28.8%, 17.2%, 18.6%, and 33.6% of the associations, respectively (all *P* for mediation <.001). Specifically, eGDR-related indices reduced the prevalence of MAFLD by lowering the levels of the A: G ratio.

### 3.5. ROC curves of IR-related indices

**Figure 3.**
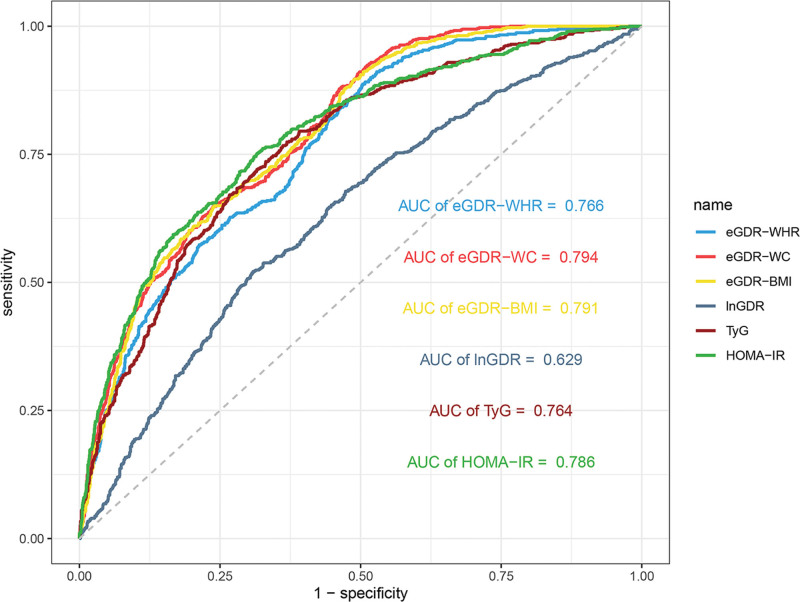
Receiver operating characteristic curves of IR-related indices in relation to MAFLD. AUC = area under curve, BMI = body mass index, eGDR = estimated glucose disposal rate, HOMA-IR, homeostasis model assessment-IR, IR = insulin resistance, lnGDR = the glucose disposal rate of the natural logarithm, MAFLD = metabolic-associated fatty liver disease, TyG = triglyceride-glucose, WC = waist circumference, WHR = waist-hip ratio.

The ROC curves of eGDR-related indices, TyG index, and HOMA-IR predicting the prevalence of MAFLD are shown in Table 4 and Figure 3. The ROC curves showed that among all the eGDR-related indices, eGDR-WC and eGDR-BMI had greater diagnostic efficacies for MALFD, their areas under the ROC curves were 0.794 (95% CI, 0.776–0.813) and 0.791 (95% CI, 0.772–0.810), respectively. Compared with TyG index and HOMA-IR, the eGDR-WC (0.794 vs 0.764 vs 0.786, *P* <.001) and eGDR-BMI (0.791 vs 0.764 vs 0.786, *P* <.001) showed better diagnostic validity for MALFD. The sensitivity and specificity for eGDR-WC were 0.881 and 0.534, respectively. The sensitivity and specificity for eGDR-BMI were 0.650 and 0.760, respectively.

**Table 4:** ROC curves analysis on the association between eGDR-related indices, TyG index and HOMA − IR and MAFLD prevalence.

Indicators	Best thresholds	Sensitivity	Specificity	AUC (95% CI)	*P* for difference
HOMA-IR	2.760	0.751	0.684	0.786 (0.766–0.807)	Reference
TyG	8.569	0.774	0.631	0.764 (0.744–0.785)	**<.001**
eGDR-WHR	9.848	0.887	0.497	0.766 (0.746–0.786)	**<.001**
eGDR-WC	9.004	0.881	0.534	0.794 (0.776–0.813)	**<.001**
eGDR-BMI	6.662	0.650	0.760	0.791 (0.772–0.810)	**<.001**
lnGDR	−5.307	0.521	0.691	0.629 (0.605–0.654)	**<.001**

Bold values indicate statistically significant differences (*P* < .05).

AUC = area under curve, BMI = body mass index, CI = confidence interval, eGDR = estimated glucose disposal rate, HOMA-IR = homeostasis model assessment of insulin resistance, lnGDR = the glucose disposal rate of the natural logarithm, MAFLD = metabolic-associated fatty liver disease, OR = odds ratio, ROC = receiver operating characteristic, TyG = triglyceride-glucose index, WC = waist circumference, WHR = waist-hip ratio.

### 3.6. Stratified analysis and sensitivity analysis

Stratified analysis by sex, age, race, poverty income ratio, education level, smoking status, activity level, and alcoholic status confirmed consistent results with the main findings (Fig. S2–5, Supplemental Digital Content, https://links.lww.com/MD/Q547). The results indicated a consistent association between eGDR-related indices and the prevalence of MAFLD across most demographic groups (*P* for interaction >.05). The negative association between eGDR-related indices and MAFLD prevalence was more likely to occur among individuals with higher education, and regular activities.

The sensitivity analysis results were in line with our primary findings. After additional adjustments for hypertension, diabetes, and cholesterol respectively, the eGDR-related indices remained significantly associated with MAFLD prevalence. In addition, the association remained robust even after transforming continuous variables into categorical variables (Table S3, Supplemental Digital Content, https://links.lww.com/MD/Q547).

## 4. Discussion

In this study, we analyzed the NHANES 2017 to 2018 dataset to explore the association between eGDR-related indices and MAFLD. Our findings indicated a negative relationship between eGDR-related indices and MAFLD, eGDR-WC and eGDR-BMI were more strongly associated with MAFLD according to estimated effect sizes. The eGDR formula’s focus on population-level metabolic assessment, rather than individual clinical diagnosis, is a critical consideration for interpreting our findings. By prioritizing reproducibility across diverse populations, this approach ensures consistency in large-scale studies like NHANES, even if it diverges from guideline-specific thresholds for hypertension. Additionally, we found that the A: G ratio, a surrogate for adipose distribution, mediated this relationship. Our analysis of ROC curves showed that eGDR-WC and eGDR-BMI had greater diagnostic validity for MAFLD among all the eGDR-related indices, and these 2 indices outperformed the TyG index and HOMA-IR in predicting MAFLD prevalence. These results highlighted the importance of eGDR-related indices in accurately identifying MAFLD patients and provided further evidence for the use of IR markers in clinical practice.

MAFLD incorporates a significant proportion globally, consistent with earlier findings, our data indicated that the prevalence of MAFLD in the general population was 36%.^[38]^ Therefore, precise identification would improve the management of this population. Individuals with MAFLD are often accompanied by IR, which can impact liver lipid deposition by regulating lipid metabolism.^[39]^ As a representative measure of IR, HOMA-IR, and TyG index have been generally regarded as potential screening indicators for MAFLD. The HOMA-IR, calculated based on fasting glucose and insulin, has been included in the diagnostic criteria of MAFLD since 2020 (29). Additionally, Wang et al carried out a meta-analysis and discovered that the TyG index could accurately diagnose and predict MAFLD patients.^[40]^ Although no studies have explored the relationship between eGDR and MAFLD, previous researches have demonstrated a significant association between eGDR levels and the prevalence of NAFLD.^[26,41]^ Similarly, compared to individuals without MALFD, individuals with MAFLD were more likely to have lower eGDR-related indices in our research. To our knowledge, our study is the first epidemiologic study with a large sample size investigating the relationship between eGDR-related indices and MAFLD prevalence.

HOMA-IR is calculated using fasting insulin, which limits the application in the general population because fasting insulin is typically measured in diabetic patients.^[42]^ Similarly, the TyG index only incorporates fasting glucose and triglycerides, neglecting other factors closely related to IR and MAFLD, such as central obesity and hypertension.^[43]^ eGDR incorporates body size, HbA1c levels, and hypertension status, all routinely assessed upon admission of people with MAFLD, hence, it is particularly suitable for early diagnosis of MAFLD. Recently, Song J et al revealed that eGDR had a superior predictive ability for all-cause mortality and cardiovascular mortality in people with NAFLD, compared to the TyG index and HOMA-IR.^[44]^ The superior predictive ability of eGDR on hepatic steatosis was also discovered in a cross-sectional study.^[45]^ Consistent with previous studies, our study explored the relationship between IR markers and MAFLD, revealing that eGDR-WC and eGDR-BMI had superior diagnostic validity than the TyG index and HOMA-IR. Compared to NAFLD, MAFLD emphasizes the roles of diabetes, hypertension, and obesity in the onset and progression of the disease.^[31]^ These factors can be assessed using eGDR-WC and eGDR-BMI, highlighting their biological significance in managing MAFLD. Therefore, merely using HOMA-IR and TyG index for diagnosing MAFLD may be insufficient, greater attention should be given to eGDR.

Abnormal distribution of adipose is closely related to MALFD.^[46]^ Our study found that eGDR-related indices were negatively associated with the A: G ratio, which effectively reflects the body adipose distribution. The mechanistic pathway between IR and adipose distribution is complex, there may be several explanations. First, insoluble collagen Iα2 is a major structural protein in connective tissues. A study from Yale found it was associated with abdominal triglycerides synthesis, insulin signaling, and body adipose distribution, revealing that IR may regulate fat distribution by influencing insoluble collagen Iα2.^[47]^ Second, insulin can regulate the postprandial energy flux of peripheral metabolic organs, promoting the absorption of glucose into peripheral organs, and preventing excessive energy storage in the visceral adipose tissue.^[48,49]^ Also, the different adipose distributions due to IR could further be determined by differential autonomic innervation of subcutaneous and visceral adipose.^[48]^ Additionally, our study revealed an important role of the A: G ratio in the relationship between eGDR-related indices and MAFLD. This finding was in line with a study of patients with diabetes, the A: G ratio was positively correlated with the prevalence of fatty liver, highlighting its crucial value for assessing the risk of hepatic steatosis.^[50]^ Moreover, this value is irreplaceable compared to other adipose distribution parameters, such as trunk adipose percent.^[51]^ This relationship between adipose distribution and MALFD has been demonstrated in the “overflow hypothesis,” which posits that the capacity to expand adipocyte size and number in subcutaneous tissue is limited, causing excess lipids to accumulate in less adapted tissues, particularly the liver, resulting in hepatic steatosis.^[13]^ Moreover, genetic and molecular studies have provided mechanistic insights. For example, the PNPLA3 rs738409 variant.^[52]^ not only affects hepatic triglyceride content but also interacts with IR to amplify lipid overflow to the liver. Similarly, XBP1-mediated ER stress may synergize with IR to disrupt adipose tissue function, further promoting visceral fat accumulation and hepatic lipid deposition.^[53]^ These mechanisms underscore the importance of adipose distribution markers like the A:G ratio in MAFLD risk stratification.

In addition, the stratified analyses indicated a significantly different effect between eGDR-related indices and MAFLD across different education levels, with a stronger negative correlation observed in individuals with higher education. MAFLD may be influenced by educational background, as individuals with higher education levels are likely more health-conscious.^[54]^ We argue that those with higher education levels are more inclined to improve IR to prevent MAFLD. Similarly, the difference was also observed in participants with different activity levels. Previous studies have shown an inverse relationship between physical activity and the A/G ratio.^[55]^ We assume that exercise may influence MAFLD prevalence through modulation of A/G ratio.

Our study had several strengths. Utilizing a complex multi-stage probability sampling method, we extracted a substantial sample from the NHANES database, ensuring accurate representation of the noninstitutionalized population and enhancing the external generalizability of our findings. More importantly, we used ROC curves to compare the diagnostic validity of the IR-related indices, indicating that eGDR-WC and eGDR-BMI performed better than the TyG index and HOMA-IR in diagnosing MAFLD. However, there were limitations to consider. Firstly, observational studies may be subject to self-reported recall bias. Secondly, as our data were limited to the U.S. population, our results may be biased and not applicable to other demographic groups. Due to the limitations inherent in cross-sectional studies, additional validation through evidence-based methodologies – such as randomized controlled trials and longitudinal research – is necessary to establish causal relationships and track MAFLD progression using advanced imaging (e.g., MRI-PDFF). Furthermore, the lack of persuasiveness of single-database data underscores the need for multi-center studies integrating diverse global datasets (e.g., UK Biobank, EPIC) to confirm the generalizability of eGDR-WC/BMI across populations. Mechanistic studies should also explore molecular pathways linking adipose distribution (A:G ratio) and IR, including genetic interactions (e.g., PNPLA3 rs738409) and collagen Iα2’s role in lipid overflow. Finally, clinical trials testing interventions (e.g., exercise, dietary changes) targeting adipose redistribution could refine eGDR-WC/BMI as actionable tools for MAFLD prevention, thereby strengthening their translational value in precision medicine.

## 5. Conclusion

These findings suggest a potential negative association between eGDR-related indices and MAFLD prevalence, with body adipose distribution (reflected by the A:G ratio) playing a mediating role. The combination of eGDR with anthropometric measures (e.g., WC or BMI) may offer improved diagnostic performance for MAFLD compared to existing indices like the TyG index or HOMA-IR. However, given the cross-sectional nature of this study, these results should be interpreted with caution, as the design precludes causal inference and may be subject to residual confounding. Clinically, the observed associations highlight the potential of eGDR-related indices as supplementary tools for early MAFLD risk identification, particularly in populations where routine metabolic and anthropometric data are readily available. Future research is critical to validate these findings in longitudinal cohorts, establish standardized cutoffs for clinical use, and evaluate the cost-effectiveness of integrating eGDR into existing diagnostic algorithms for MAFLD.

## Acknowledgments

The authors thank the participants and investigators of the NHANES databases.

## Author contributions

**Formal analysis:** Rong He, Kecen Zhao.

**Investigation:** Rong He, Kecen Zhao.

**Project administration:** Kecen Zhao.

**Supervision:** Kecen Zhao.

**Writing – original draft:** Rong He.

**Writing – review & editing:** Kecen Zhao.

## Supplementary Material


