# Supplementary material for: Association between estimated glucose disposal rate and metabolic-associated fatty liver disease among US adults: A cross-sectional study
Source: Medicine (Baltimore). 2025 Oct 31;104(44):e45652. doi: 10.1097/MD.0000000000045652 (PMC12582842; doi:10.1097/MD.0000000000045652)

Supplementary Table 1

Baseline characteristics of excluded and included data

**Characteristic**	**Excluded Data**, N = 2,305 [a]	**Included Data**, N = 2,273 [a]	**Standardized Difference (%)** [b]
Age (%)			
<65 years	1,821 (82%)	1,854 (85%)	8
≥65 years	484 (18%)	419 (15%)	8
Sex (%)			
Female	1,171 (50%)	1,105 (50%)	0
Male	1,134 (50%)	1,168 (50%)	0
Race/Ethnicity (%)			
Mexican American	360 (12%)	324 (9%)	9
Other/multiracial	1,945 (88%)	1,949 (91%)	9
BMI (kg/m^2)			
<25.0	934 (39%)	592 (27%)	25
Overweight	674 (31%)	726 (30%)	2
Obesity	697 (30%)	955 (43%)	27
Activity level (%)			

**Characteristic**	**Excluded Data**, N = 2,305 [a]	**Included Data**, N = 2,273 [a]	**Standardized Difference (%)** [b]
No activities	915 (49%)	1,065 (41%)	16
Regular activities	770 (51%)	1,208 (59%)	16
Smoking status (%)			
Never smoker	1,093 (61%)	1,251 (57%)	8
Smoker	592 (39%)	1,022 (43%)	8
Hypertension (%yes)	944 (39%)	1,150 (44%)	10
Diabetes (%yes)	338 (11%)	342 (11%)	0

[a]median (IQR) for continuous; n (%) for categorical

[b]The difference between the groups divided by the pooled standard deviation; a value higher than 10% is interpreted as a meaningful difference.

Supplementary Table 2

The relationship between eGDR-related indices and A: G ratio.

	**Continuous OR (95% CI)** [b]	**Q1** [c] **OR (95% CI)**	**Q2 OR (95% CI)**	**Q3 OR (95% CI)**	**Q4 OR (95% CI)**	P_{trend} [e]
eGDR-WHR [a]	0.96 (0.96, 0.97)	Ref [d](1.00)	0.94 (0.87, 1.01)	0.87 (0.81, 0.93)	0.77 (0.73, 0.81)	**<0.001**
eGDR-WC	0.96 (0.96, 0.97)	Ref [d](1.00)	0.93 (0.86, 1.01)	0.91 (0.84, 0.98)	0.76 (0.72, 0.79)	**<0.001**
eGDR-BMI	0.97 (0.96, 0.97)	Ref [d](1.00)	0.93 (0.85, 1.01)	0.91 (0.85, 0.98)	0.77 (0.73, 0.81)	**<0.001**
lnGDR	0.97 (0.96, 0.98)	Ref [d](1.00)	1.00 (0.91, 1.09)	0.93 (0.85, 1.02)	0.89 (0.84, 0.95)	**0.001**

[a]Model was adjusted for age, gender, and race, family income, activity level, educational level, alcoholic status, and smoking status.

[b]Data were listed as the weighted odd ratio estimates and 95% confidence intervals.

[c]Q, quartile.

[d]Ref, reference.

[e]Tests for trends based on the variables containing the median values for each quartile.

eGDR, estimated glucose disposal rate; A: G ratio, android: gynoid ratio; WHR, waist-hip ratio; WC, waist circumference; BMI, body mass index; lnGDR, the glucose disposal rate of the natural logarithm; OR, odds ratio; CI, confidence interval.

Supplementary Table 3

Sensitivity analysis of of the relationship between eGDR-related indices and MAFLD.

		MAFLD		
		**OR (95% CI) [b]**	**Effect sizes**	**P value**
Model 4 [a]	eGDR-WHR	0.32 (0.25, 0.42)	-1.13	< **0.001**
	eGDR-WC	0.39 (0.33, 0.45)	-0.95	< **0.001**
	eGDR-BMI	0.42 (0.35, 0.50)	-0.87	< **0.001**
	lnGDR	0.84 (0.69, 1.02)	-0.17	0.064
Model 5	eGDR-WHR	0.67 (0.59, 0.77)	-0.40	**0.002**
	eGDR-WC	0.63 (0.57, 0.70)	-0.46	< **0.001**
	eGDR-BMI	0.63 (0.57, 0.69)	-0.46	< **0.001**
	lnGDR	0.76 (0.65, 0.90)	-0.27	**0.013**
Model 6	eGDR-WHR	0.65 (0.57, 0.73)	-0.43	**0.002**
	eGDR-WC	0.62 (0.55, 0.68)	-0.49	< **0.001**
	eGDR-BMI	0.62 (0.56, 0.68)	-0.48	< **0.001**
	lnGDR	0.77 (0.66, 0.91)	-0.26	**0.016**
Model 7	eGDR-WHR	0.66 (0.59, 0.73)	-0.41	**0.001**
	eGDR-WC	0.62 (0.57, 0.69)	-0.47	< **0.001**
	eGDR-BMI	0.62 (0.56, 0.68)	-0.48	< **0.001**
	lnGDR	0.74 (0.63, 0.87)	-0.30	**0.010**

[a]Based on Model 3, hypertension (yes or no), diabetes (yes or no), and cholesterol were added into the multivariable logistic regression model for additional adjustment in Model 4-6, respectively. In Model 7, continuous variables (age and income) were transformed into categorical variables based on Model 3.

[b]Data were listed as the weighted odd ratio estimates and 95% confidence intervals.

MAFLD, metabolic-associated fatty liver disease; eGDR, estimated glucose disposal rate; WHR, waist-hip ratio; WC, waist circumference; BMI, body mass index; lnGDR, the glucose disposal rate of the natural logarithm; OR, odds ratio; CI, confidence interval.

Supplementary Figure 1. Flowchart of sample selection in NHANES.

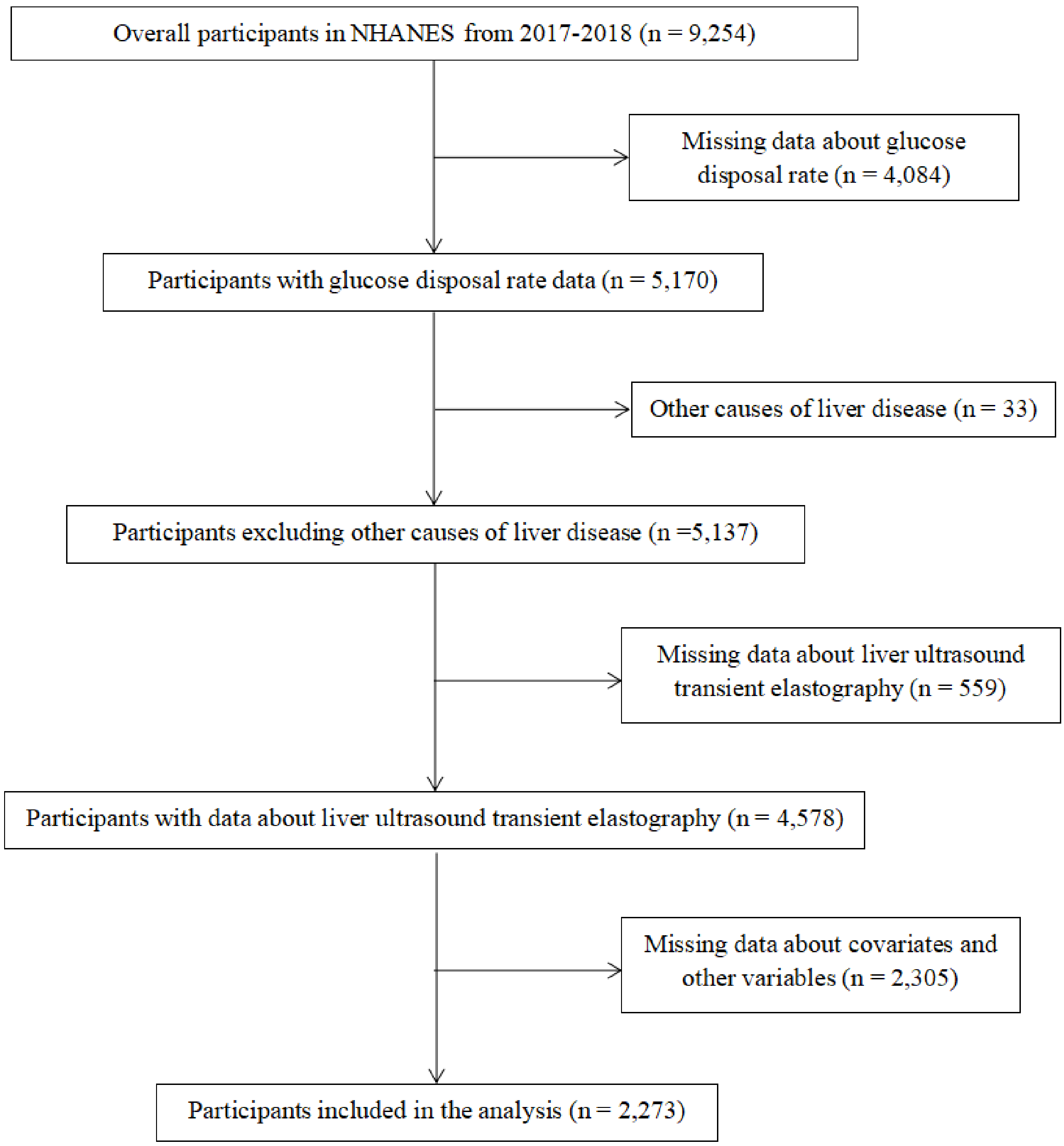

Supplementary Figure 2. Forest plots for subgroup analysis of the relationship between eGDR-WHR and MAFLD. Subgroup analysis was stratified by age, sex, race, poverty income ratio, education level, smoking status, activity level, and alcoholic status. OR, odds ratio; CI, confidence interval; MAFLD, metabolic-associated fatty liver disease; eGDR, estimated glucose disposal rate; WHR, waist-hip ratio.

Subgroup	No.of participants	OR (95% CI)	P for interaction
**Sex**			**0.127**
Male	1168	0.61 (0.51,0.73)	
Female	1105	0.69 (0.60,0.79)	
**Age**			**0.984**
<60 years	1594	0.64 (0.56,0.73)	
≥60 years	679	0.66 (0.56,0.78)	
**Race/ethnicity**			**0.648**
Mexican American	324	0.66 (0.38,1.16)	
Non-Hispanic White	848	0.65 (0.57,0.74)	
Non-Hispanic Black	500	0.64 (0.57,0.72)	
Other/multiracial	601	0.60 (0.51,0.70)	
**Poverty-to-income ratio**			**0.052**
Low level	563	0.64 (0.57,0.73)	
Middle level	918	0.68 (0.58,0.79)	
High level	792	0.62 (0.52,0.74)	
**Education level**			**0.036**
<High school	324	0.75 (0.60,0.93)	
≥High school	1949	0.64 (0.58,0.70)	
**Smoking status**			**0.801**
Never smoker	1251	0.66 (0.56,0.79)	
Smoker	1022	0.62 (0.54,0.71)	
**Activity level**			**0.186**
No activities	1065	0.68 (0.59,0.78)	
Regular activities	1208	0.61 (0.52,0.70)	
**Alcoholic status**			**0.551**
No-excessive alcohol	1169	0.64 (0.58,0.72)	
Excessive alcohol	1104	0.64 (0.55,0.76)	

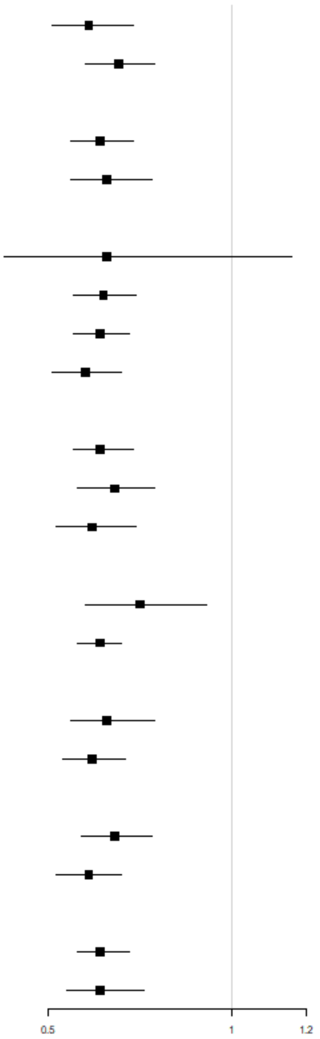

Supplementary Figure 3. Forest plots for subgroup analysis of the relationship between eGDR-WC and MAFLD. Subgroup analysis was stratified by age, sex, race, poverty income ratio, education level, smoking status, activity level, and alcoholic status. OR, odds ratio; CI, confidence interval; MAFLD, metabolic-associated fatty liver disease; eGDR, estimated glucose disposal rate; WC, waist circumference.

Subgroup	No.of participants	OR (95% CI)	P for interaction
**Sex**			**0.120**
Male	1168	0.57 (0.50,0.65)	
Female	1105	0.65 (0.57,0.75)	
**Age**			**0.798**
<60 years	1594	0.61 (0.55,0.69)	
≥60 years	679	0.62 (0.51,0.76)	
**Race/ethnicity**			**0.752**
Mexican American	324	0.62 (0.37,1.04)	
Non-Hispanic White	848	0.61 (0.55,0.69)	
Non-Hispanic Black	500	0.63 (0.55,0.73)	
Other/multiracial	601	0.59 (0.51,0.68)	
**Poverty-to-income ratio**			**0.058**
Low level	563	0.63 (0.54,0.72)	
Middle level	918	0.64 (0.54,0.73)	
High level	792	0.58 (0.51,0.67)	
**Education level**			**0.146**
<High school	324	0.66 (0.53,0.82)	
≥High school	1949	0.61 (0.56,0.66)	
**Smoking status**			**0.823**
Never smoker	1251	0.62 (0.52,0.73)	
Smoker	1022	0.61 (0.55,0.67)	
**Activity level**			**0.029**
No activities	1065	0.66 (0.59,0.73)	
Regular activities	1208	0.57 (0.51,0.64)	
**Alcoholic status**			**0.593**
No-excessive alcohol	1169	0.61 (0.54,0.69)	
Excessive alcohol	1104	0.62 (0.54,0.70)	

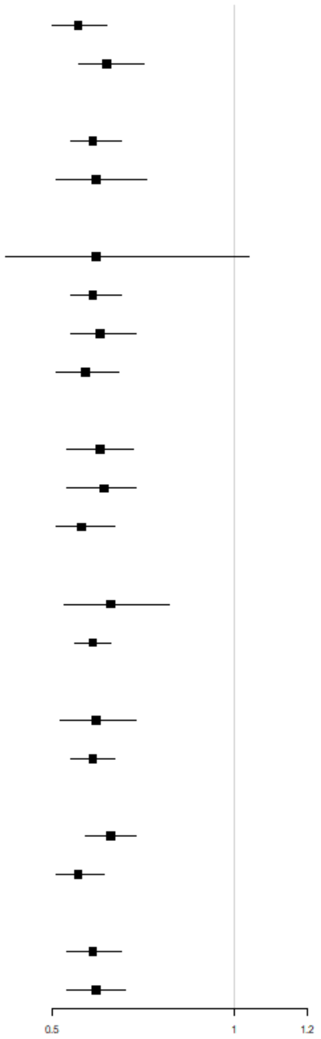

Supplementary Figure 4. Forest plots for subgroup analysis of the relationship between eGDR-BMI and MAFLD. Subgroup analysis was stratified by age, sex, race, poverty income ratio, education level, smoking status, activity level, and alcoholic status. OR, odds ratio; CI, confidence interval; MAFLD, metabolic-associated fatty liver disease; eGDR, estimated glucose disposal rate; BMI, body mass index.

Subgroup	No.of participants	OR (95% CI)	P for interaction
**Sex**			**0.092**
Male	1168	0.57 (0.50,0.65)	
Female	1105	0.65 (0.57,0.75)	
**Age**			**0.980**
<60 years	1594	0.61 (0.55,0.68)	
≥60 years	679	0.62 (0.51,0.76)	
**Race/ethnicity**			**0.785**
Mexican American	324	0.61 (0.36,1.03)	
Non-Hispanic White	848	0.61 (0.56,0.68)	
Non-Hispanic Black	500	0.64 (0.55,0.74)	
Other/multiracial	601	0.59 (0.51,0.69)	
**Poverty-to-income ratio**			**0.041**
Low level	563	0.64 (0.55,0.73)	
Middle level	918	0.64 (0.56,0.73)	
High level	792	0.57 (0.50,0.66)	
**Education level**			**0.020**
<High school	324	0.67 (0.54,0.84)	
≥High school	1949	0.61 (0.56,0.66)	
**Smoking status**			**0.995**
Never smoker	1251	0.62 (0.53,0.73)	
Smoker	1022	0.60 (0.54,0.68)	
**Activity level**			**0.844**
No activities	1065	0.65 (0.59,0.72)	
Regular activities	1208	0.57 (0.52,0.63)	
**Alcoholic status**			**0.670**
No-excessive alcohol	1169	0.61 (0.54,0.70)	
Excessive alcohol	1104	0.62 (0.55,0.69)	

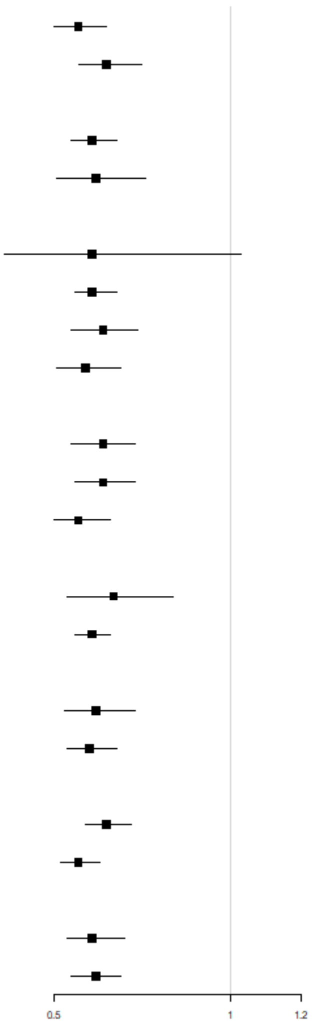

Supplementary Figure 5. Forest plots for subgroup analysis of the relationship between lnGDR and MAFLD. Subgroup analysis was stratified by age, sex, race, poverty income ratio, education level, smoking status, activity level, and alcoholic status. OR, odds ratio; CI, confidence interval; MAFLD, metabolic-associated fatty liver disease; lnGDR, the glucose disposal rate of the natural logarithm.

Subgroup	No.of participants	OR (95% CI)	P for interaction
**Sex**			**0.095**
Male	1168	0.70 (0.55,0.88)	
Female	1105	0.83 (0.73,0.96)	
**Age**			**0.194**
<60 years	1594	0.73 (0.61,0.88)	
≥60 years	679	0.81 (0.59,1.10)	
**Race/ethnicity**			**0.221**
Mexican American	324	0.75 (0.53,1.05)	
Non-Hispanic White	848	0.73 (0.61,0.89)	
Non-Hispanic Black	500	0.72 (0.57,0.91)	
Other/multiracial	601	0.86 (0.70,1.07)	
**Poverty-to-income ratio**			**0.328**
Low level	563	0.72 (0.61,0.86)	
Middle level	918	0.78 (0.63,0.98)	
High level	792	0.73 (0.56,0.94)	
**Education level**			**0.006**
<High school	324	0.95 (0.73,1.23)	
≥High school	1949	0.74 (0.64,0.86)	
**Smoking status**			**0.718**
Never smoker	1251	0.77 (0.63,0.95)	
Smoker	1022	0.77 (0.64,0.91)	
**Activity level**			**0.020**
No activities	1065	0.79 (0.68,0.93)	
Regular activities	1208	0.72 (0.59,0.83)	
**Alcoholic status**			**0.351**
No-excessive alcohol	1169	0.72 (0.58,0.90)	
Excessive alcohol	1104	0.79 (0.66,0.94)	

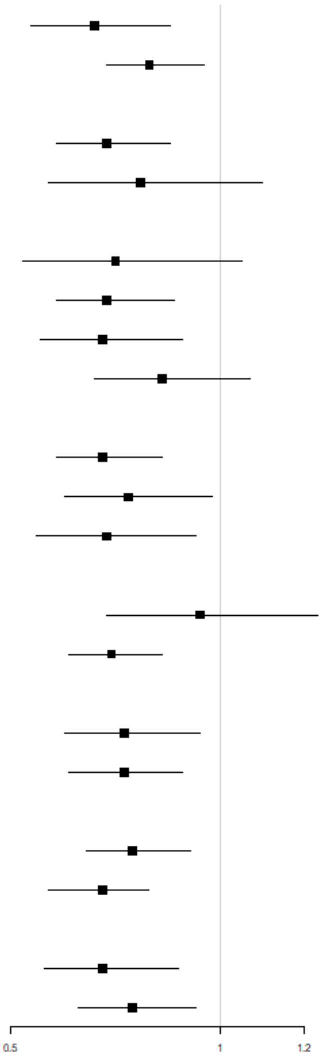

Supplement: Supplementary file 1 [file medi-104-e45652-s001.pdf]
